# Genome-Wide Prediction Methods in Highly Diverse and Heterozygous Species: Proof-of-Concept through Simulation in Grapevine

**DOI:** 10.1371/journal.pone.0110436

**Published:** 2014-11-03

**Authors:** Agota Fodor, Vincent Segura, Marie Denis, Samuel Neuenschwander, Alexandre Fournier-Level, Philippe Chatelet, Félix Abdel Aziz Homa, Thierry Lacombe, Patrice This, Loic Le Cunff

**Affiliations:** 1 UMT Geno-Vigne, IFV-INRA-Montpellier Supagro, Montpellier, France; 2 UMR AGAP, INRA, Montpellier, France; 3 UR 588 AGPF, INRA, Orléans, France; 4 UMR AGAP, CIRAD, Montpellier, France; 5 University of Lausanne, Department of Ecology and Evolution, Lausanne, Switzerland; 6 University of Lausanne, Swiss Institute of Bioinformatics, Vital-IT, Lausanne, Switzerland; 7 Department of Genetics, The University of Melbourne, Parkville, Australia; Memorial Sloan Kettering Cancer Center, United States of America

## Abstract

Nowadays, genome-wide association studies (GWAS) and genomic selection (GS) methods which use genome-wide marker data for phenotype prediction are of much potential interest in plant breeding. However, to our knowledge, no studies have been performed yet on the predictive ability of these methods for structured traits when using training populations with high levels of genetic diversity. Such an example of a highly heterozygous, perennial species is grapevine. The present study compares the accuracy of models based on GWAS or GS alone, or in combination, for predicting simple or complex traits, linked or not with population structure. In order to explore the relevance of these methods in this context, we performed simulations using approx 90,000 SNPs on a population of 3,000 individuals structured into three groups and corresponding to published diversity grapevine data. To estimate the parameters of the prediction models, we defined four training populations of 1,000 individuals, corresponding to these three groups and a core collection. Finally, to estimate the accuracy of the models, we also simulated four breeding populations of 200 individuals. Although prediction accuracy was low when breeding populations were too distant from the training populations, high accuracy levels were obtained using the sole core-collection as training population. The highest prediction accuracy was obtained (up to 0.9) using the combined GWAS-GS model. We thus recommend using the combined prediction model and a core-collection as training population for grapevine breeding or for other important economic crops with the same characteristics.

## Introduction

Thanks to new sequencing technologies (NGS), use of molecular markers is nowadays much less expensive, allowing the development of genome-wide approaches for characterizing the genetic architecture of complex traits, or for marker assisted selection, such as genome-wide association studies (GWAS) or genomic selection (GS).

Recently, GWAS has been widely used in plant genetics to understand genetic architecture and identify molecular polymorphisms explaining part of the variation for traits of agricultural interest [1]–[3]. These markers can then be used in marker-assisted selection (MAS) programs. GWAS has identified many common alleles of major effect, however it is less efficient to detect associations for structured traits [4], [5]. Indeed, traits of agricultural interest may be correlated with environmental gradients and lead to confounding effects in association tests. In a similar way, the impact of human selection may also strengthen population structure, all the “elite” breeds sharing a narrow genetic base, thus leading to false positives (type II errors) in association tests. Moreover the efficiency of GWAS is also impacted by the genetic architecture of the studied trait: indeed, the detection of linked molecular markers in polygenic traits strongly depends both on the size of the sample and on the density of molecular marker used [6]–[8].

Genomic selection (GS) is a more recent methodology to make a more efficient use of whole genome information in MAS. In contrast to GWAS methodology which identifies molecular polymorphisms linked to the variation for selected traits, GS allows the prediction of a breeding value – genomic estimated breeding values (GEBV) – for the genotypes tested [9] based on large sets of markers. Previous studies on animal and plant models, based on both simulated and real data, demonstrated the interest of GS, especially for capturing small-effect quantitative trait loci [10]–[14]. In breeding programs, GS could significantly reduce costs by limiting both size and number of field experiments and by facilitating early selection through an efficient use of molecular information. Genotype-based prediction also allows selection in breeding schemes when the phenotyping of breeding candidates is impossible or difficult [15]–[18].

In GS, as the number of markers greatly exceeds the number of individuals, advanced statistical methods are definitely required. In recent years, many different methods were developed to realize these predictions (reviewed and compared in [17], [19], [20]). To take into account a large variety of genetic architectures, some models assume that all genomic segments equally affect phenotype, whereas others assume heterogeneity among SNP effects and consider different shapes of the prior distribution for marker effects (Bayesian approaches).

Today, most studies have concentrated on animal models or annual plants, with large pedigrees or complex breeding schemes. However, in several economically important species, such as coffee, orange and grapevine, this type of information and breeding material are not available (no pre-breeding population) due to the biological characteristics of these crops. Grapevine is one of the earliest domesticated fruit crops [21] that has been widely cultivated for its fruits and wine. Studying molecular data of a very large set of *Vitis vinifera* L. subsp. *vinifera*, [22] identified three groups of varieties based on their geographical origin and their use. The most commonly acknowledged scenario [23]–[26] dates grape domestication back to circa 5,000 years BC in the Eastern Caspian region (primary domestication center). Through selection, mostly targeted at large-sized, clear-colored berries and hermaphrodite flowers, a coherent sub-population emerged (denoted “Table-East”, TE). Due to human migrations, domesticated varieties were introduced in the Balkans around 4,000 BC where they crossed with local wild individuals and were then selected for small berries to produce wine, forming the group denoted “Wine-East” (WE) group [22]. Finally viticulture arrived in Western Europe around 1,000 BC and wine varieties from the Balkans crossed with local wild individuals forming the “Wine-West” (WW) group.

In grapevine, no advanced breeding lines from complex schemes are available. Instead, breeders are handling a large parental panel with a high diversity both at morphological and molecular level. This material is highly heterozygous (He  = 0.76) [27], as a result of a strong inbreeding depression and the predominance of vegetative propagation which maintained a high level of molecular diversity [27]–[30]. This panel is also characterized by a low level of linkage disequilibrium (LD) between marker loci (r^2^∼0.2 at 5-10 Kb) [29], [30]. Most cultivars are interconnected by a series of first-degree relationships (for example, Pinot noir – Chardonnay – Gouais blanc, Cabernet franc – Merlot [31], [32]), but the number of connected generations is rather low [33], [34]. Furthermore some major agricultural traits (for example berry size) are linked to population structure, making association studies difficult [35].

Since the demand for new grapevine cultivars with sustainable resistance/tolerance traits and well adapted to climate changes is increasing [36]–[38], and since the number of molecular tools available for this species is soaring, GWAS and GS are indeed becoming relevant in this crop. The first set of high density genome-wide molecular markers, developed on eight *Vitis* species comprised 9K SNP (Vitis9KSNP array) and was successfully used for preliminary assessment of germplasm collections [30]. A new 18K genotyping chip is already available [39] but will only increase the number of markers available for *Vitis vinifera* L. up to 20K. Because of the rapid decay of LD observed in grapevine [30] hundreds of thousands of markers would be necessary to perform efficient GWAS and GS. Such number would only be reached by resequencing hundreds of cultivars. Since developing the resources enabling marker-assisted selection at the whole genome level in grape will still require heavy work, it is indispensable to perform a preliminary assessment of the feasibility of MAS, targeting structured or unstructured traits using GS in a broad pool of unrelated genetic resources. This will allow testing the limitations and potential uses of GWAS and GS in grapevine through simulated data sets.

In this work we simulated genomic and phenotypic data for a large set of individuals to obtain highly polymorphic, heterozygous, structured populations similar to the present population of cultivated *Vitis vinifera* L. Using these virtual populations, we performed both GWAS and GS for traits of different complexity using a large set of markers compatible with the extent of LD in this species. The objectives were i) to test GWAS ability to detect simulated quantitative trait loci ii) to analyze and to compare the performance of a prediction based on markers identified through GWAS (classic MAS) with all marker using GS methods iii) and to estimate the influence of trait complexity and structure on prediction accuracy, using different combination of training and candidate sets defined in a structured population.

## Materials and Methods

### Simulation

We simulated a population of 3,000 individuals representing the genetic diversity of *Vitis vinifera* L., based on the knowledge presently available on the history of this species [22]–[27], [34], [40], [41].

Simulated genomes comprised the typical 19 chromosomes, each of 79 cM, for a total of 1,500 cM corresponding to the genetic map of grapevine published by [42]. Ten thousand markers were randomly positioned on each chromosome, for a total of 189,500 bi-allelic markers (SNP), and 500 multi-allelic markers (SSR, 20 alleles per locus) with a mutation rate of 10e-6 and 10e-4 per generation, respectively [43], [44]. Considering that genome length in grapevine is 470 Mb [45], one simulated cM corresponds to 300 Kb. We simulated four independents quantitative traits: i) structured simple trait (10 QTL), ii) non-structured simple trait (10 QTL), iii) structured complex trait (100 QTL), iv) non-structured complex trait (100 QTL, under the assumption of strict additivity. QTLs were bi-allelic loci, randomly positioned on the genome. One of the two possible alleles had an effect of zero (no effect on the trait), while the other had an effect randomly sampled from a normal distribution (with mean  = 0 and variance = 1).

**Figure 1:**
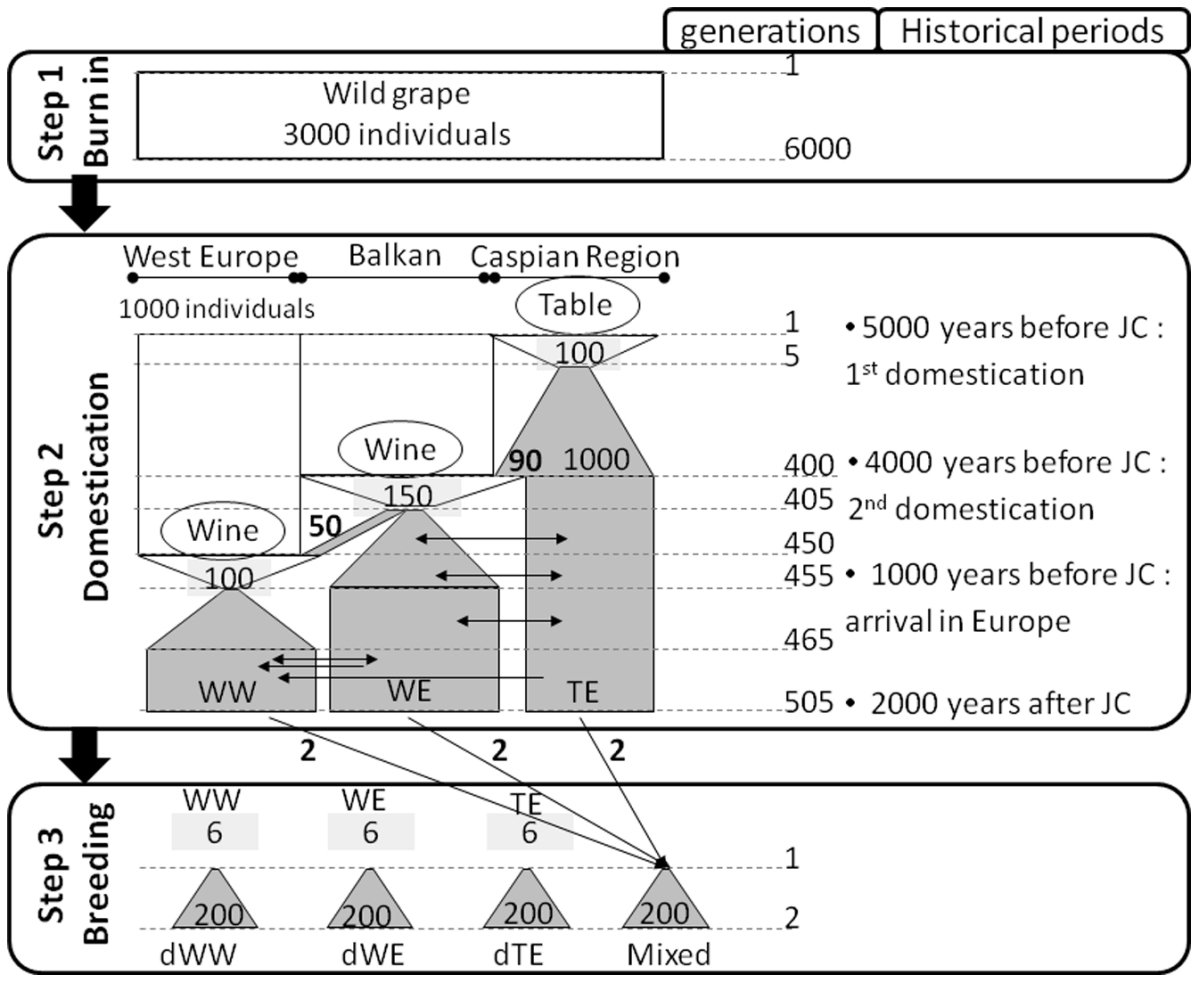
Scheme of the demographical scenario based on our working hypothesis on grapevine evolution. This scheme, implemented with quantiNemo, is composed of three steps: burn-in, domestication and breeding. Burn-in and domestication steps had the purpose to obtain grapevine diversity groups corresponding to Western Europe wine group (WW), Eastern Europe and Balkan wine group (WE) and Eastern Europe and Caucasus table group (TE) as described by [22]. Breeding step models crosses between selected individuals of these groups. At the right side of the figure are represented generation numbers and historical events with dates. White area is representing wild grape, after domestication it is showed grey. “Wine” and “Table” symbolize the two different definitions of selection applied on the trait under selection (selection optima and intensity). Black arrows show the direction of migration and its intensity is indicated by boldface numbers, specifying the number of migrating individuals. The stringency of each bottleneck is indicated by specifying the number of selected individuals (in regular font).

Simulations were carried out with a modified version of quantiNEMO, an individual-based program developed for the analysis of quantitative traits with explicit genetic architecture potentially under selection in a structured population [46]. We based our demographic scenario (Figure 1) on grapevine domestication history and our goal was to define a scenario matching the published population data (F_ST_, LD, heterozygosity and population structure; [22], [27], [30], [47]. This demographic scenario consisted in two steps (burn-in and domestication) to obtain presently existing material and a third step (breeding) to simulate a breeding program.

In order to simulate a wild, pre-domestication population with realistic allele frequencies and LD between neutral loci at mutation-drift equilibrium, we ran a burn-in step as a common starting point for the ten replicates of the domestication step. A single population was simulated with a census population size and carrying capacity of 3,000. It was run for 6,000 generations with random mating to obtain the required LD level (r^2^ value of 0.2 observed at the distance of 10 kb) between neutral markers and to generate enough segregating sites for the following analyses. At the end of the burn-in step, fixed loci were removed and individuals were randomly organized in three groups (sub-populations) of 1,000 individuals, forming a meta-population.

Step 2 consisted in the domestication step. It was established to obtain the three diversity groups of the cultivated compartment of *Vitis vinifera* L. subsp. *vinifera* described by [22] in the Vassal collection: the “Table-East” group (TE) corresponding to the table grape varieties originated from the primary domestication center, localized in the Caucasus, the “Wine-East” group (WE) of wine varieties from the Balkans and Eastern Europe, and the “Wine-West” group (WW) of wine varieties from Western and Central Europe.

It is difficult to estimate the number of generations throughout grape domestication history as grape is a long-lived perennial species. Propagation type varied greatly between vegetative and generative methods at different times and in the different grapevine-growing areas. Based on historical data and personal communication by J.M. Boursiquot and T. Lacombe, we chose to run the domestication step for about 500 generations. Simulating 505 generations allowed recreating a population structure (F_ST_ and structure) and linkage disequilibrium (LD) pattern similar to what is currently observed in cultivated grape.

The migration rate between each pair of population was set to vary over time in order to fit to historical information and to obtain the required heterozygosity and F_ST_ between populations at the end of the domestication step. To justify the choice of the migration rates we tested alternative scenarios varying these values between no migration and twice more important migration rate. The size of the bottleneck at the beginning of the domestication was calibrated in the same way, using alternative scenarios without bottleneck and with a bottleneck twice more stringent than in the finally chosen scenario.

Using the same demographic parameters we elaborated two versions with different quantitative trait architectures: simple (quantitative trait controlled by 10 QTLs) and complex (quantitative trait controlled by 100 QTLs) following [10] and [48]. To simulate quantitative traits linked to population structure, we applied stabilizing selection for the first quantitative trait with both levels of complexity. Intensity and optima of selection varied among populations (to simulate different selection objectives) and over time (time since the selection bottleneck). The genetic architecture of a quantitative trait under selection affects genetic diversity evolution at the sub-population level. In order to maintain the same F_ST_ and to generate similar Q_ST_ (as a measure of phenotypic differentiation among population) for both complexity levels we adjusted the intensity and the optimum of the stabilizing selection in each domestication scenario. The heritability of quantitative traits was set by fixing the environmental variance to achieve a narrow-sense heritability of 0.8 in the first generation of the simulation.

Finally, we added a breeding step, simulating crosses between and within sub-populations, to mimic the effects of a breeding program. Founding individuals were chosen from each of the three sub-populations based on their phenotypic value for the trait under selection. For within sub-populations crosses, we chose the six individuals with the best phenotypic record compared to the selection optimum. For between sub-populations crosses we used the two individuals closest to the phenotypic mean of each sub-population of origin. In this way, we obtained four populations with six individuals in each, producing four times 200 descendants in the next generation via random mating. No selection and migration were used in this final step. Simulated genotypic and phenotypic data for one replicate of the three original populations and the breeding populations are available in File S1 in Information S1.

### Core collection

MSTRAT software (v 4.1) developed by [49] used the M-method proposed by [50] and allowed the construction of core collections that maximize the number of observed alleles in the SSR data set. We defined a core-collection from the meta-population of 3,000 individuals using MStrat software and the 500 SSRs. This core-collection (Call) consisted in 1,000 individuals, including the founders of all breeding populations; it was built to represent the genetic diversity of the entire meta-population (all) with minimal redundancy (which is the aim concept of core-collection building). In each replicate of the domestication step, five core collections of 1,000 individuals were designed and ranked first by the number of SSR alleles captured; core-collections exhibiting the same allelic richness (determined by the total number of alleles represented) were then ranked using Shannon's index as second criterion. Finally, the core-collection presenting the most significant allelic richness with the highest Shannon's index was selected for further analysis.

### Estimation of diversity indices

Diversity indices, such as genetic variance estimates, the level of differentiation in quantitative trait (Q_ST_) following [51], and F-statistics following [52] for each pair of populations and for all types of markers, were calculated with quantiNemo. To calculate unbiased heterozygosity and compare it to published data [27] on highly polymorphic SSR markers, we selected all SSR with more than 10 alleles per locus at the end of the domestication step. Data analysis was performed using the “Excel Microsatellite Toolkit” [53]. We also calculated allele frequency for each SNP and QTL locus, in order to filter out rare SNPs with minor allele frequency (MAF) below 5% that would have biased association tests.

### Population structure and relatedness

Population structure was calculated on the 3,000 individuals using 500 SSR with STRUCTURE software version 2.3.3 [54] accessed through Bioportal [55]. We used an admixture model varying the ancestral number of population (K) from two to five, in order to identify the best K level of population subdivision. Within STRUCTURE, we allowed an iterative process with a burn-in phase of 15,000 iterations and a sampling phase of 15,000 replicates. Five replicates of each assumed K level subdivision were compared to estimate group assignation stability. Outputs were visualized and interpreted with Structure Harvester web v0.6.93 [56]. The optimal group number was chosen based on the estimated ‘log probability of data’.

Realized relationship matrix (RRM; [57] was calculated using R [58] using all filtered SNPs (MAF>5%)on 3,000 individuals.

### Linkage disequilibrium

LD measures were performed with the R package LDcorSV [59] which corrects for the bias due to population structure and relatedness (r^2^_SV_). LD was measured in two different positions: in neutral genomic regions and around each QTL. In neutral positions, mean and median values of r^2^ were calculated between each pair of SNP within five arbitrarily chosen windows of 600 kb. Around QTLs, r^2^ was calculated between the QTL locus and all SNP located within 300 kb. We used the Hill and Weir formula [60] for describing the decay of r^2^_SV_ and we characterized LD by the distance corresponding to a r^2^_SV_ value of 0.2.

### Genome-wide association

GWAS were performed using the multi-locus mixed-model (mlmm) approach [61], including the population structure as fixed covariant in the mixed model. This R script implements a forward-backward stepwise approach to include significant effects in the mixed model, while re-estimating the variance components of the model at each step. We ran mlmm on the meta-population of 3,000 individuals and on the core-collection with a random polygenic term, with a variance proportional to the estimated RRM and a fixed population structure term (three groups) consisting in ancestry fractions estimated by Structure software. We also ran mlmm on each sub-population with a random polygenic term only. Maximal number of forward steps was set to 25. For model selection we chose the multiple-Bonferroni (mBonf) criterion, selecting the largest model in which all cofactors have a P-value below a Bonferroni-corrected threshold (we used a threshold of 0.05). Cofactor effects were re-estimated at the end of the mlmm analysis and used to estimate the genetic value of descendent obtained in the breeding step in the simulation.

### Genomic prediction

We compared four prediction methods based on genome-wide high density SNP data: the sum of effects of markers previously detected in GWAS – using mlmm as described above – corresponding to classical MAS (cof), Ridge Regression BLUP (RR) [62], Bayesian LASSO (Least Absolute Shrinkage and Selection Operator) Regression (BLR) [63] and a combination of MAS and RR-BLUP (cofRR). We also observed the evolution of prediction accuracy in different combinations of training and candidate populations. Training population always comprised 1,000 individuals, while candidate populations were composed of 200 or 800 individuals. We compared two levels of genetic architecture (10 or 100 underlying QTLs) and prediction accuracy of structured and non-structured quantitative traits (design summarized in Figure S1 in Information S1).

For cof method, effects of significant markers and populations structure were first estimated with a mixed-model together with variances for genetic (polygenic) and residual random effects. In this model the groups of population structure and the significant markers were declared as fixed effects. Then, in a second step the estimates of the associated markers were used for prediction.

Ridge Regression performs an extent of shrinkage that is homogenous across markers. For RR we defined the parameter lambda as 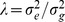, where environmental and genetic variances (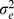 and 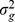) were estimated via REML in a mixed linear model using emma library [64].

The Bayesian LASSO [65] method performs stronger shrinkage toward zero for the estimates of small-effect markers, and less for those with high effects. We performed BLR analysis with the R package BLR 1.3 [63]. The lambda parameter was set as random, sampled from a gamma distribution with rate = 0.0001 and shape = 0.53 [65]. The initial value of λ_0_ was calculated using the heritability rules given in [20]: 
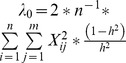
 where h^2^ is the narrow-sense heritability, n is the number of individuals, m is the number of SNPs and X is the matrix of genotypes. 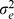 were chosen from the prior 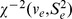, where 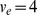 to ensure a finite a priori variance, and 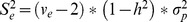, where 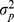 is the phenotypic variance. 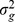 were chosen from the prior 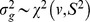 where 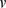 was 4 to ensure a finite a priori variance and 
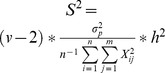
. We allowed an iterative process with a burn-in phase of 10,000 iterations and a sampling phase of 40,000 replicates.

In marker-assisted RR (cofRR) we combined RR-BLUP with the effects of markers previously detected with mlmm. Effects of significant markers and population structure were estimated as described for cof method and remaining SNPs were used in a RR model as described earlier. GEBVs were obtained summing the effects of all markers. The R script is available in File S2 in Information S1. Accuracy was calculated dividing the correlation coefficient (r^2^) between GEBVs and true phenotypes, by the square root of the narrow-sense heritability.

### Test on pine data

The method cofRR was tested on a real data set of loblolly pine described in [66] using a 10-fold cross-validation schema. Data consisted of 926 individuals genotyped with 4,853 SNPs and phenotyped for 17 traits. Information about population structure was not available.

For the analysis, markers with more than 20% of missing data were removed in both training and validation sets. For the remaining loci, missing genotypes were imputed with the mean. In the training set, we applied a filtering of 5% on minor allele frequency (MAF>0.05). Kinship matrix (RRM) was calculated as described above. GWAS were performed using mlmm approach setting the maximal number of forward steps to 10. To limit the detection of false-associated cofactors, we choose the extended Bayesian information criterion (EBIC [67]) for model selection, which is more stringent than the multiple Bonferroni criterion [61]. Predictions were performed using cof, RR and cofRR methods as described previously.

For the 10-fold cross-validation, individuals were randomly assigned to one of 10 equal folds. Each fold was dropped once from the training set and predicted. Accuracies were calculated as described above and using the Mendelian segregation as heritability according to [66], and the mean value was reported across all 10 folds.

## Results

### Simulation

We built the demographic scenario to simulate *Vitis vinifera* L. history in order to create three genetic pools as observed by [22]. Parameters (migration rate and bottleneck) of the domestication step were defined from bibliographic data. In order to validate the chosen migration rate and bottleneck intensity, we also tested four alternative scenarios i) without migration, ii) with a twice higher migration rate, iii) without bottleneck and iv) with a twice more stringent bottleneck. Ten replicates of each scenario were simulated. Diversity indices (F_ST_, Q_ST_, heterozygosity) were calculated for all five scenarios and compared to published data. The values obtained with the domestication step were closer to the expected level than for the alternative scenarios (Table 1). Heterozygosity was the only parameter with a value lower than expected (0.64 vs. 0.73), being closer to the level observed in natural populations of *Vitis sylvestris* [27]. Changing bottleneck and migration ratio modified all diversity indices.

**Table 1:** Population statistics on simulated data for the five scenarios and reference values from published data.

		Domestication step	Published data	Alternative scenarios			
				No migration	Twice more migration	No bottleneck	Double intensity bottleneck
FST	WW-WE	0.04 (0.007)	0.05^a^	0.34 (0.018)	0.01 (0.001)	0.01 (0.001)	0.05 (0.012)
	WW-TE	0.07 (0.012)	0.07^a^	0.35 (0.001)	0.01 (0.003)	0.03 (0.003)	0.09 (0.015)
	WE-TE	0.04 (0.007)	0.05^a^	0.45 (0.014)	0.01 (0.001)	0.03 (0,001)	0.04 (0.008)
Heterozygosity		0.64 (0.026)	0.73^b^	0.46 (0.011)	0.64 (0.019)	0.72 (0.005)	0.60 (0.012)

The standard deviation is between brackets.

a [47],

b [27].

### Descriptive statistics on simulated data

Because of genetic drift and selection, the number of polymorphic loci decreased over time. While, at the beginning of the burn-in step (common to the 10 replicated simulations), 189,500 polymorphic SNP loci were defined, 111,004 polymorphic SNP loci only were observed at the end of this step (Table 2). After 505 generations, at the end of the domestication step, we observed on average 92,787 (sd  = 309.5) polymorphic SNP loci for the entire meta-population of 3,000 individuals. After filtering on minor allele frequency (MAF>0.05) 81,555 SNPs (sd  = 845.6) were retained. For both simple and complex quantitative traits (confounded or not with demographic structure) on average 85% of the QTLs were polymorphic and 73% passed the MAF>0.05 filter.

**Table 2:** Descriptive statistics on the simulated meta-population.

simple trait	complex trait	Real
LD		11 kb		10^a^
SNP number	Total	111,004		-
	polymorphic	92,787.1 (309.5)		-
	MAF>0.05	81,555.0 (845.6)		-
QTL number	Total	2×10	2×100	-
	polymorphic	8.6 (1.03)	83.7 (3.94)	-
	MAF>0.05	7.2 (1.51)	72.2 (4.72)	-
heritability	structured trait	0.71 (0.080)	0.76 (0.037)	-
	Non-structured trait	0.78 (0.034)	0.77 (0.025)	-

a [30].

**Figure 2:**
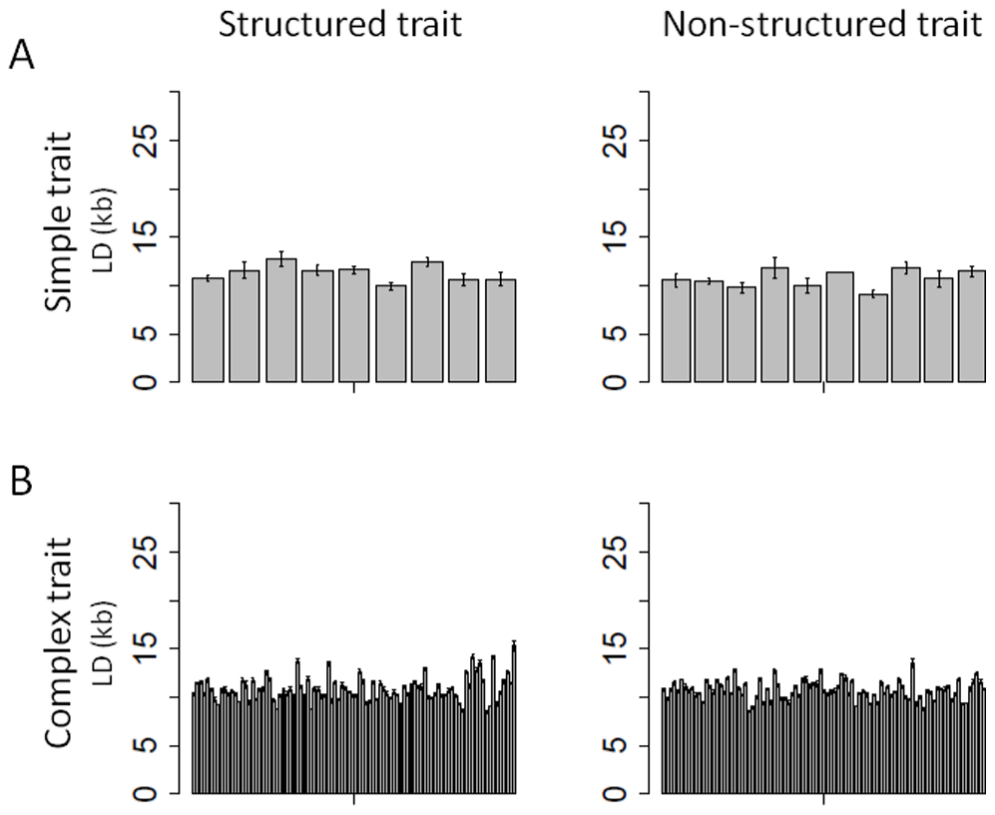
Estimation of LD around QTLs. Mean estimation of LD (in Kb) around the QTLs, calculated at r^2^SV  = 0.2 between all loci in the 600 Kb neighborhood of each QTL locus on 3,000 individuals, for simple traits (**A**) and complex traits (**B**) on the 10 replicates of the simulation. The two figures on the left side represent LD around structured trait's QTLs and the other two figures around non-structured traits QTLs. QTL loci were ranked as a function of theirs effects from negative to positive values. Error bars were calculated with 95% confidence intervals on the estimates of the means.

We measured LD decay in both neutral genomic regions and around QTLs. LD in neutral regions decreased rapidly (Figure S2 in Information S1). An r^2^_SV_ value of 0.2 was observed over a distance of nine to 13 kb depending on the replicate. This value is consistent with the LD observed over 10 kb segments in a set of grape cultivars [30]. Around QTLs, we observed the same tendency except for structured traits, where LD extended further than 13 Kb in a few cases (Figure 2). Consequently, given the extent of LD, the number of SNPs present at the end of the domestication step allowed us to tag all the genome.

The F_ST_ statistics between simulated populations were measured with SSR markers. As expected from observed data [47] the historically more distant populations (WW-TE) showed the highest F_ST_ values of 0.07 while historically closer populations displayed lower (approx. 0.04) F_ST_ values (Table 1, Figure S3 in Information S1).

The Structure analysis (L(K) method) over the entire meta-population (3,000 individuals) best supported clustering into three ancestral populations in all replicates of the simulation (data not shown) corresponding to the expected three simulated populations: WW, WE and TE.

The narrow-sense heritabilities for the simulated traits at the end of the domestication step were approx. 0.8 (0.72 to 0.78 for simple trait and 0.76 to 0.77 for complex) conform to initial settings. Q_ST_ was measured as an index of phenotypic distances between each pair of simulated sub-population. Q_ST_ values were always higher for selected traits than for neutral ones (Figure S3 in Information S1). Overall Q_ST_ values reflected F_ST_ values with the TE population diverging more from the other two populations. However, since no published data on Q_ST_ are available yet, we were unable to compare our data with actual observations.

In conclusion, the simulated populations matched observed data reasonably well. We thus considered that the demographic scenario was able to generate pertinent genotypic and phenotypic data allowing further GWA studies and the building of GS models.

### Descendent populations

**Figure 3:**
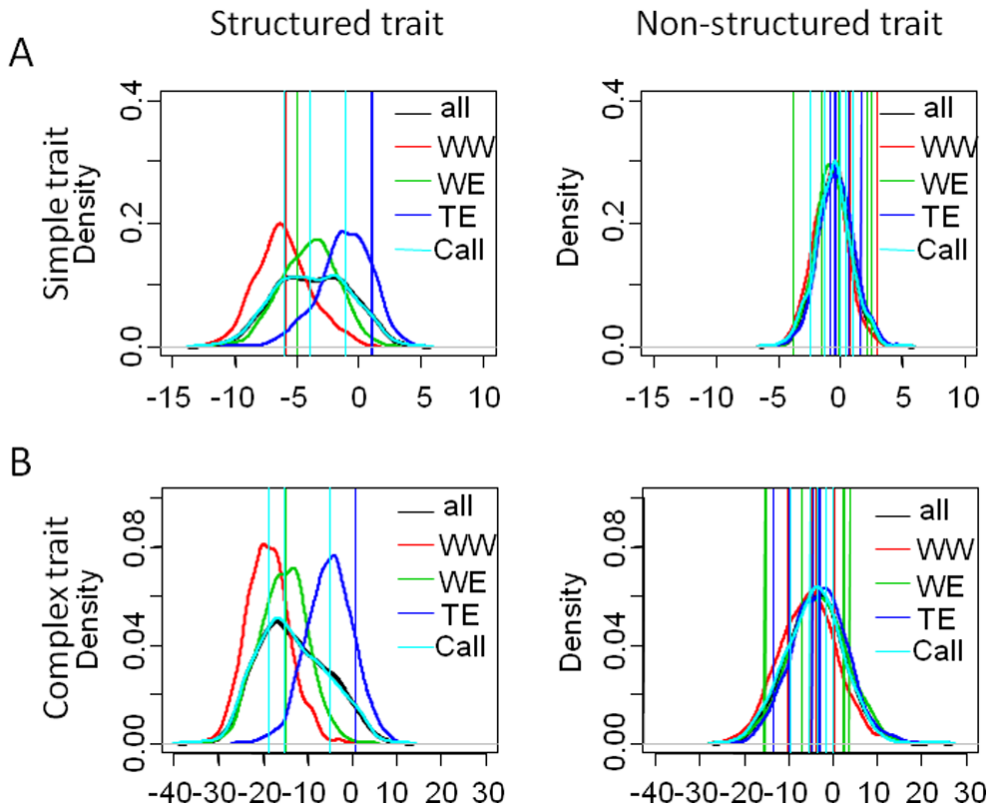
Distribution of phenotypes in training (WW, WE, TE) populations. Distributions are presented on one replicate of the simulation for the structured and non-structured simple (**A**) and complex (**B**) traits. The colored vertical lines show the phenotypes of the founder individuals of descendent populations. Call corresponds to the core-collection.

To simulate a breeding program, we crossed selected individuals from the three original gene pools (Figure 1). Three crosses were realized within populations leading to dWW, dWE, dTE, and one between populations leading to Mixed. In the original gene pools, traits distributions for non-structured traits were identical between sub-populations while they were different for the structured traits (Figure 3). Variance for simple traits was also smaller than for complex traits.

**Figure 4:**
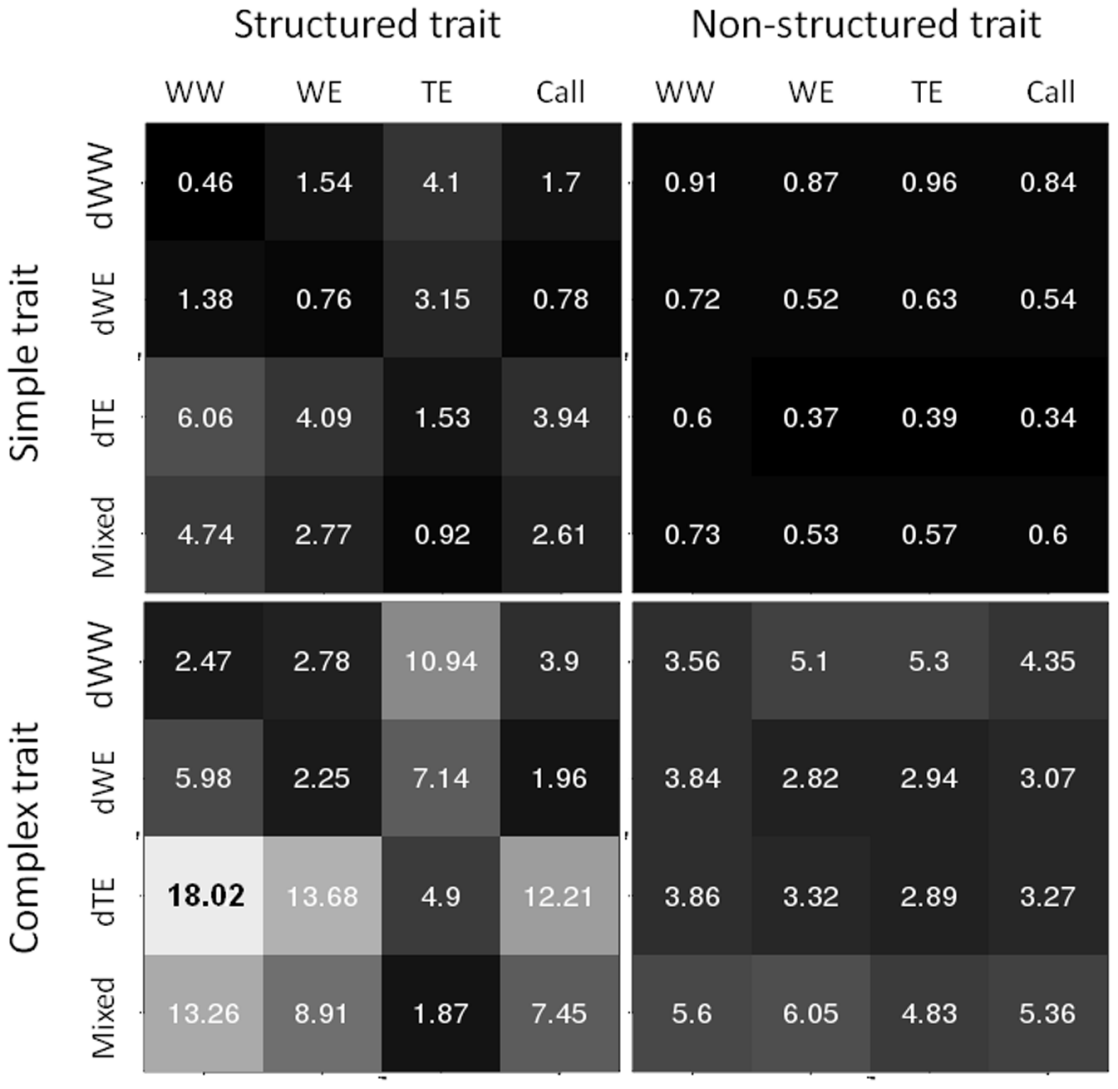
Heat map presenting the difference between the phenotypic mean of training and candidate sets. Mean values were calculated on the 10 replicates of the simulation.

The differences between mean phenotypic values of the breeding crosses and their respective original gene pools were smaller for simple traits than for complex ones (Figure 4). It was slightly higher between WW and dWW for non-structured traits compared to the other populations, but the highest difference was obtained between TE and dTE for structured traits.

Differences in phenotypic means were also measured between the breeding crosses and i) those original gene pools without direct parental link ii) the core-collection. We observed greater differences for structured traits than for non-structured ones and for simple traits than for complex ones (Figure 4). dTE is always more distant from the other sub-populations, while Call behaves similarly to WE, and the Mixed population is closer to TE than to the other populations.

### Genome-wide association study (GWAS)

The best mlmm model of each replicate realized on the whole meta-population explained 68 to 83% of the total variance. As expected, the composition of the variance differed between simulated traits (Figure S4 in Information S1). Through the 10 replicates of the simulation of the four training sets (WW, WE, TE, Call, i.e. 1,000 individuals), significant associations were detected for 32 to 59% (on average) of the simulated QTLs in simple traits and 2 to 5% in the complex traits (Table 3). For simple traits, one to four QTL only were never detected through replicates, while for complex traits this number ranged from 77 to 88. The proportion of fixed QTLs was similar for all traits, on average 14 to 18% per replicate. Some QTLs were always fixed across the 10 replicates: one in the simple structured trait and five in complex traits. In the case of non-structured traits, one QTL was repeatedly detected across replicates for the simple trait and another QTL was detected in two subpopulations for the complex trait. As expected, more QTL could be identified for non-structured traits than in structured ones, especially with the simple trait (55 to 57%, while in non-structured trait only 32 to 37%). In the full meta-population of 3,000 individuals (all), more QTL were detected than in the training sets of 1,000 individuals, especially for complex traits. In the core-collection fewer QTL were identified than in sub-populations. Manhattan plots of the results in one replicate are shown as supplementary data (Figure S5 in Information S1). In this example, SNPs linked to QTLs were detected for all types of traits with very high P-values (Table S1 in Information S1).

**Table 3:** Results of GWAs analyses.

		Structured traits					Non-structured traits				
		through 10 replicates			mean per replicate		through 10 replicates			mean per replicate	
		never detected	always fixed	always detected	fixed	detected	never detected	always fixed	always detected	fixed	detected
Simple trait	WW	40%	10%	10%	14%	32%	10%	0%	10%	15%	59%
	WE	30%	10%	10%	16%	37%	20%	0%	10%	15%	57%
	TE	40%	10%	10%	16%	32%	10%	0%	10%	14%	57%
	Call	40%	10%	10%	14%	32%	10%	0%	10%	14%	55%
	all	40%	10%	10%	14%	39%	10%	0%	10%	14%	69%
Complex trait	WW	84%	5%	0%	17%	3%	84%	5%	1%	17%	5%
	WE	77%	5%	0%	17%	5%	86%	5%	1%	17%	5%
	TE	81%	5%	0%	17%	5%	84%	5%	0%	18%	5%
	Call	86%	5%	0%	16%	2%	88%	5%	0%	17%	4%
	all	62%	5%	0%	16%	13%	71%	5%	4%	17%	12%

This table presents the number of positive detection via associated markers of each simulated QTL using the mlmm method, out of 10 replicates for both simple and complex traits and for structured and non-structured traits.

LD measures between QTLs and the cofactors of mlmm showed that significant markers always presented higher LD with the closest QTL, than with other QTLs. However, some cofactors presented quite weak linkage (r^2^<0.05) with the QTL, but strong linkage (r^2^>0.2) with another cofactor, itself tightly linked to the QTL.

### Prediction of phenotypes from genotypes

**Figure 5:**
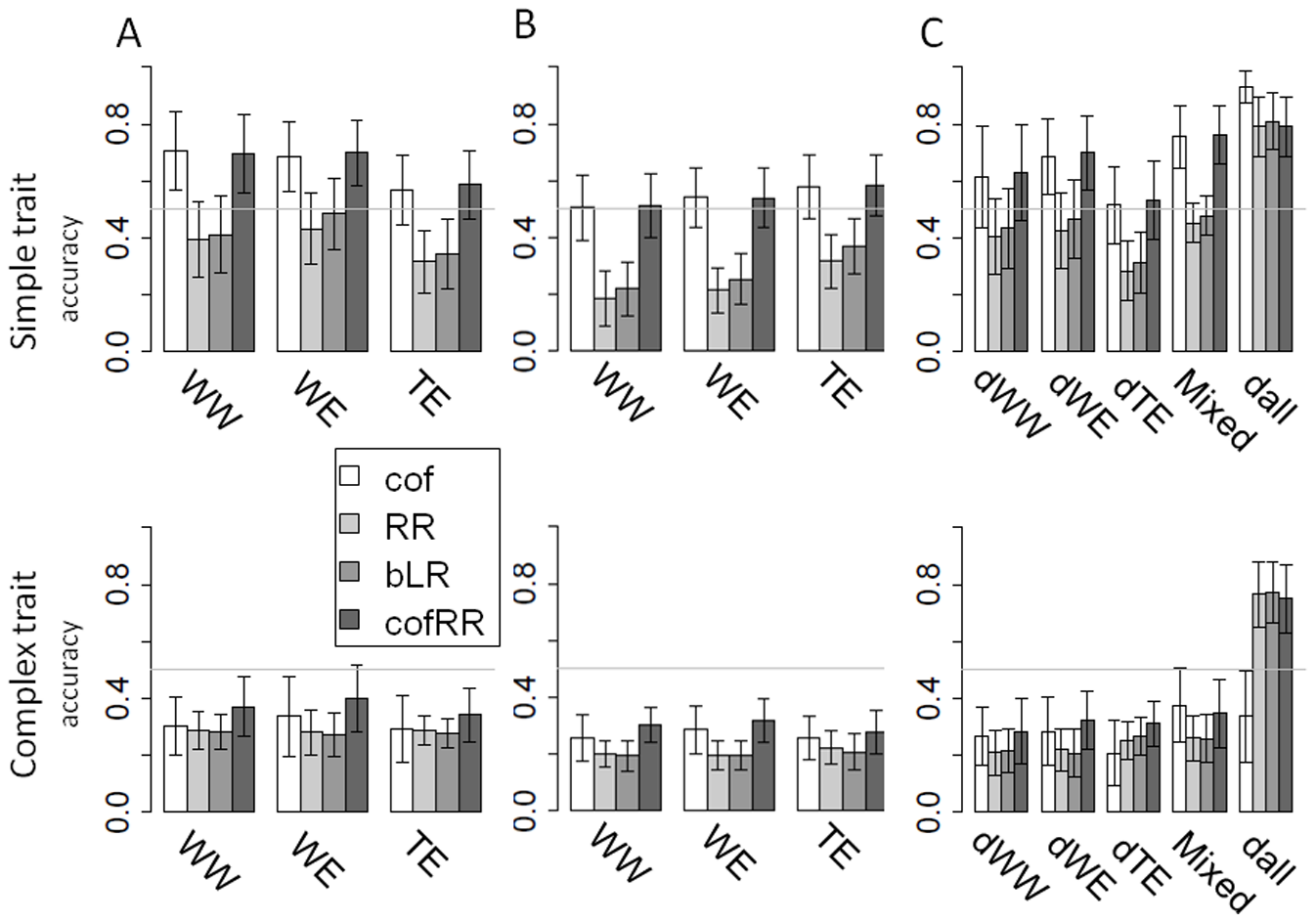
Mean prediction accuracy as a function of the training – candidate combination. Results are showed on simple and complex traits through the 10 replicates of the simulation. Figure **A** presents the prediction within sub-population (candidate set derived from the training set). Figure **B** shows the mean accuracy of prediction between sub-population (candidate sub-populations derived from a different training set). Training sets are indicated on the x axis, the four colors representing the four methods used (cof, RR, BLR, cofRR). Training and candidate sets comprised all individuals of the indicated sub-population (1,000 and 200 individuals respectively). In figure **C** the prediction models were built on the core-collection (Call) and applied to the four breeding sub-populations separately (dWW, dWE, dTE and Mixed, each composed of 200 individuals) and to the whole meta-population (dall, 800 individuals).

**Figure 6:**
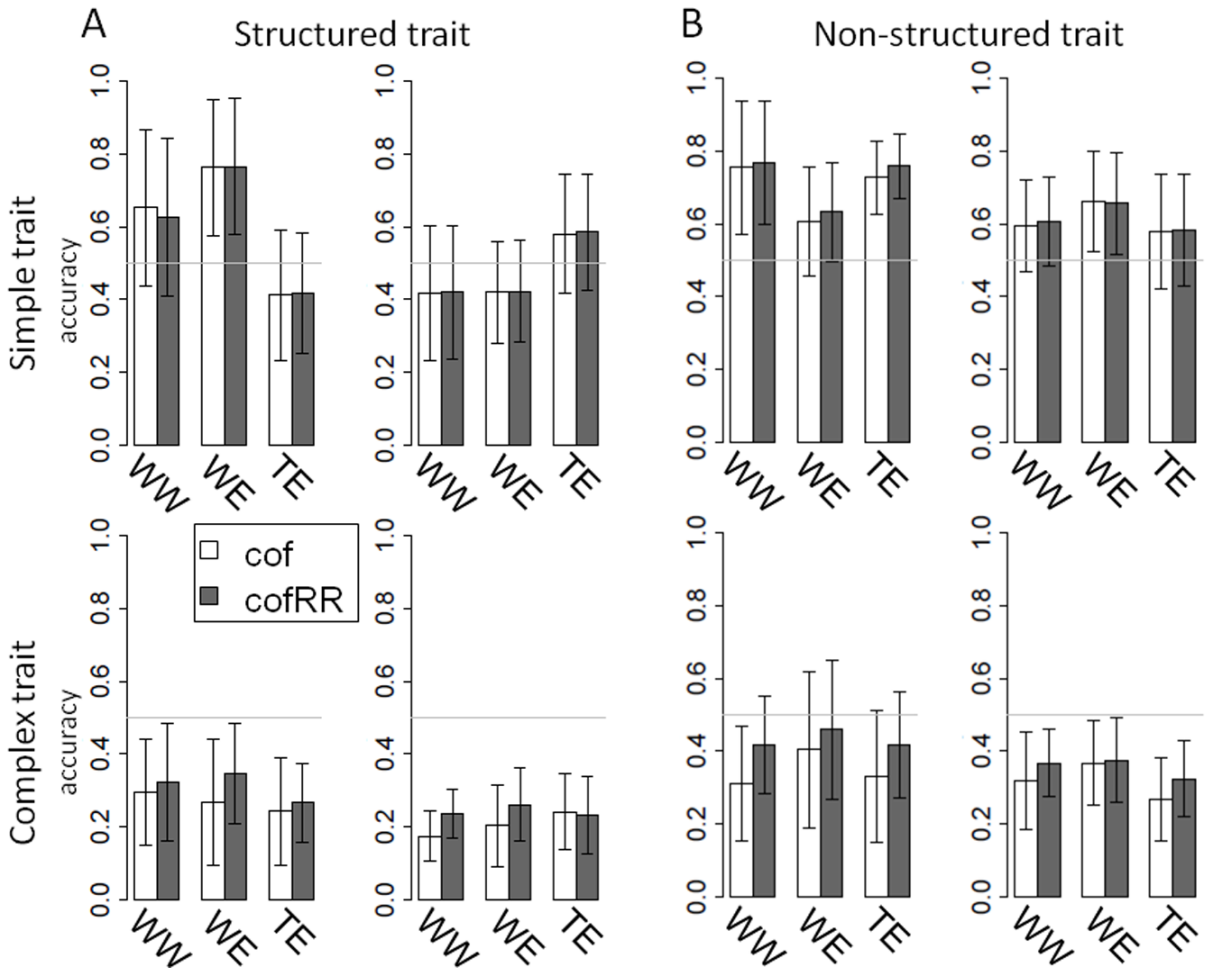
Mean accuracy of prediction in structured (A) and non-structured (B) trait. We also compared here two combinations of training – candidate sets (i.e. the two figures on the left present within sub-population predictions and the two figures on the right present between sub-population predictions) and simple and complex traits through 10 replicates of the simulation. Training sets are indicated on the x axis, the two colors representing the methods used (cof, cofRR). Training and candidate sets comprised all individuals of the sub-population (1,000 and 200 individuals respectively), except for the model constructed on Call, which was tested on the entire breeding population (800 individuals).

We used four methods (cof, RR, BLR, cofRR) to predict descendent populations phenotypes from their genotypes based on prediction models defined on the training populations (Figure S6 in Information S1). We tested different combinations of training versus candidate populations in order to compare their prediction power in different situations of relationship and for different trait complexities and structures (Figure 5–6).

#### Model selection

Auto-prediction (candidate set  =  training population) with high accuracy proved the relevance of all the models used (Figure S7 in Information S1). Globally, the prediction models showed low (0.2) to high (0.9) accuracy depending on the methods, traits and combination of training and candidate populations. Simple traits were always better predicted than complex ones (accuracy of up to 0.9 versus accuracy of up to 0.5). Models built with cof and cofRR methods always performed better than models built with the other methods for simple traits (mean accuracy on the 10 replicates of 0.2 to 0.85 versus 0.1 to 0.5; Figure S6 in Information S1). For complex traits, cof method was always as efficient as RR and BLR.

#### Relationship between training and candidate populations

As expected, accuracies obtained from within sub-population predictions were always better than between sub-population predictions (+0.3% to 400%; Figure 5A and 5B). Among within sub-populations predictions, accuracies for simple traits were better with WW and WE as training set than with TE, while no significant difference was observed for complex traits. Using the core-collection as training population, accuracies obtained on dWW, dWE and dTE were as good as for within sub-population prediction (Figure 5C). Accuracy was slightly better for the Mixed sub-population than for the others. The best accuracies were obtained predicting the totality of the descendant meta-population (800 individuals, dall). In this case cof method results showed a 15% better accuracy than other methods for simple traits, while it was 56% less accurate for complex traits.

#### The effect of trait structure

**Figure 7:**
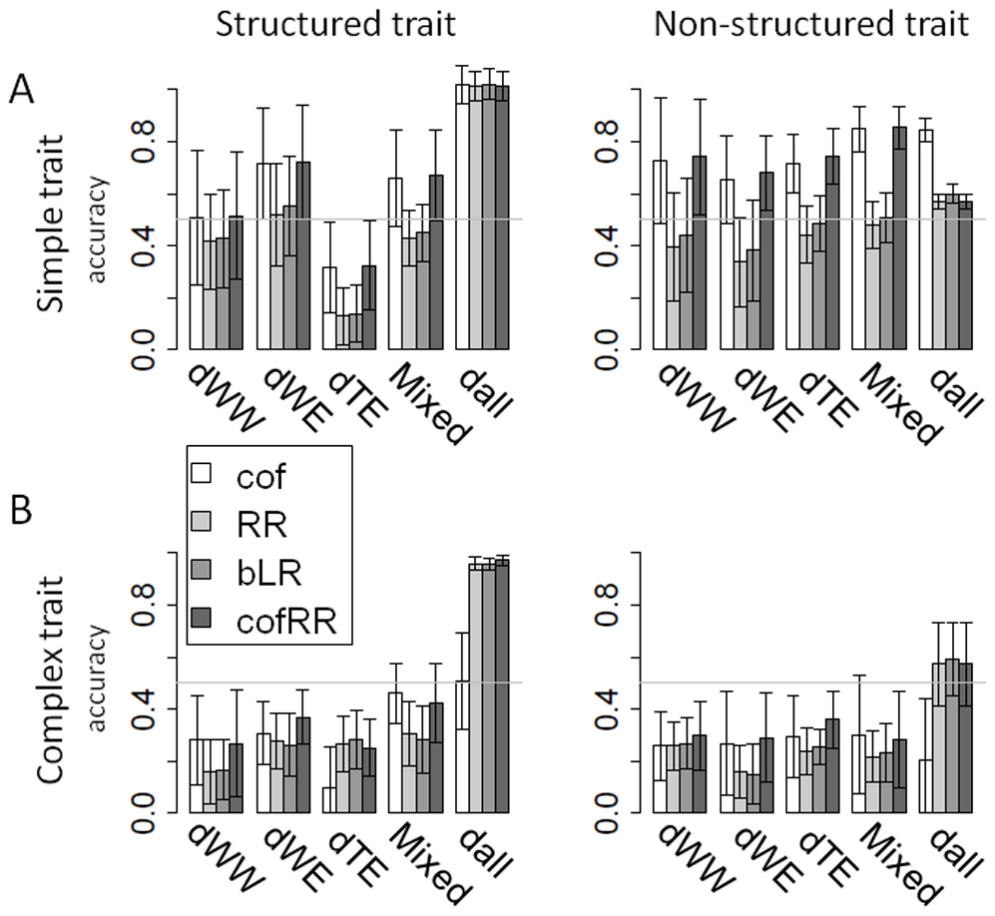
Prediction accuracy in structured (A) and non-structured (B) traits using the core-collection as training population. Mean prediction accuracy was calculated on all 10 replicates of the simulation using four methods (cof, RR, BLR, cofRR). Models were built on the core-collection (Call) and applied to the four breeding sub-populations separately (dWW, dWE, dTE and Mixed, each composed of 200 individuals) and on the whole breeding meta-population (dall, 800 individuals). The two figures on the left side represent accuracies observed on structured traits and the other two figures accuracies on non-structured traits.

Structured and non-structured traits were predicted within and between sub-population using cof and cofRR methods (Figure 6) and also with the core-collection as training set (Figure 7). We observed slightly higher values for non-structured traits than for structured traits, except in the case of WE for simple traits. All markers using models built on the core-collection predicted the structured traits better than the non-structured ones on dWE and on the entire meta-population. In these cases they highly out-performed cof method for complex traits (200 to 300%).

### Pine data

After filtering on missing data and allele frequency, around 3047 (+/−5) SNPs were considered for the GWAS. There was only one trait out of 17 (fusiform rust susceptibility by presence or absence of rust: Rust_bin) where cofactors could always be identified through the 10 training sets of the cross-validation schema. In this case, higher accuracies were obtained with cofRR method than with RR or cof. For the traits where no cofactors could be identified with mlmm, cof method accuracy was equal to zero, while RR and cofRR methods displayed the same accuracies. The supplementary Figure S8 in Information S1 presents the accuracy of these three methods on two traits having similar Mendelian segregation values (0.26 and 0.21 respectively). The first one is the average branch diameter of six years old trees (BD) considered as a complex architecture trait. No cofactor could be detected for this trait, so RR and cofRR yielded the same accuracy (0.50). The second trait is Rust_bin, an oligogenic trait, where one or two cofactors were detected depending on the training set. Cof method showed poor prediction accuracy (0.24), while cofRR resulted in an accuracy of 0.77, thus outperforming RR method (0.67).

## Discussion

### Simulated data

Because high density SNP markers (over 20 K) are still unavailable in grape, we have used simulations in order to test both GWAS and GS. Three populations of 1,000 individuals were simulated in order to reflect real data [22]: three genetic pools of high heterozygosity (He  = 0.74) but with relatively low differentiation (F_ST_ values of up to 0.07).

The simulation of genomes and causative mechanisms (genetic architecture) in different species is complex. There are many different forms of genomic variability, a wide variety of plausible demographic and evolutionary histories, as well as considerable uncertainty about how mutation and recombination rates vary and about the mode and distribution of gene action [68]. We chose a forward-simulation strategy and developed a complex demographic scenario based on historical information, which was implemented using quantiNemo software [46]. We simulated natural (Hardy-Weinberg) populations with additional human selection and migration following historical data about grapevine's domestication. Despite its early domestication, human breeding in grape seems rather recent and was not very intensive compared to other crops (maize, rice). Instead of creating advanced lines from complex breeding schemes, a large genetic diversity was maintained and is still cultivated today [33]. For unknown or hard to estimate parameters (bottleneck, migration rate, selection intensity, variation of parameters in the time, number of generations), we followed guidelines from grapevine's evolution history and defined alternative scenario to test the sensitivity of these parameters. The number of generations since grapevine's domestication was also difficult to estimate because of the combination of vegetative and generative propagation methods over time and across different geographical regions. Several sources suggested a very limited number of generative cycles. For wine cultivars Arroyo-García et al. (2006) estimated 80 generations [24], while Fournier-Level et al. (2010) expected 100 [69]. The values we used in our scenarios (505 generations for TE, 100 for WE and 50 for WW) were supported by these historical informations, with a constraint to achieve desired population structure (F_ST_ and structure) and to create linkage disequilibrium (LD) between QTLs and surrounding neutral markers.

The simulation of the meta-population based on grape evolution's history led a large set of individuals forming highly polymorphic heterozygous structured populations close to the cultivated compartment of *Vitis vinifera* L. Heterozygosity level was however a little lower than observed, closer to the natural populations of *V. sylvestris*, the wild compartment of grape, which underwent little to no human selection. In this simulated data LD level around the QTLs was slightly higher than in neutral regions of the genome (nine to 16 kb and nine to 13 kb respectively). However, more extended LD can be observed in the region of QTLs controlling binary traits, such as berry color [70] and muscat flavor [71]. Indeed, [34], using only 5,110 polymorphic SNPs on 289 individuals, were able to identify by GWAS several associations for berry color, which is a highly selected binary trait, indicating an extensive LD between loci located within a 43-kb region [70]. Nevertheless our study focused on quantitative traits, which are nowadays challenging breeding programs, and where genome-wide selection methods are needed.

In the simulations, a large number of parameters were declared (more than 50). These values were defined following the evolutionary history of grape and comparing multiple alternative scenarios. Finally we chose the model which best fitted real data based on four criteria: F_ST_, LD, heterozygosity and population structure. The scenario we developed is just one possibility to create the target material. This model could be optimized using the Approximate Bayesian Computation (ABC) approach [72], but its implementation is very time-consuming and exceeds the scope of this study.

### Feasibility of GWAS in grape

One aim of this study was to test GWAS ability to detect simulated QTLs in highly heterozygous genomes in a structured meta-population with high level of genetic diversity, similar to grapevine. Genomes were covered by more than 80,000 well-distributed SNP markers and analyses realized with the mlmm method [61]. We simulated four sets of 1,000 individuals (WW, WE, TE, Call) to investigate the genetic properties of four quantitative traits characterized by two levels of complexity (10 or 100 QTLs), linked or not to population structure.

GWAS was more efficient to detect a few QTLs with a large effect (characteristic of simple traits) than to identify multiple loci of too small additive effects, as showed in previous studies [1]. In structured and complex traits, a number of underlying QTLs could never be perceived because of fixation. Due to the confounding effect of population structure in structured traits – using a model controlling for population structure – we detected slightly fewer associations explaining a smaller part of the total variance than in non-structured traits, as already mentioned [6]–[8], [35]. In this work, we fixed the number of SNPs to 111,000 (of which 92,787 remained polymorphic after running the simulation) so that at least one to two SNPs were present in every LD block of 10 kb. The cases where QTLs could not be detected were due to the small effect (percentage of the variance explained) of these loci (Figure S9 in Information S1). Increasing the sample size of the studied panel can be a solution to detect these QTLs. Indeed, using 1,000 individuals instead of 3,000, only half of the QTLs could be identified in our data (Table S1 in Information S1). Similarly, fewer QTL were identified, especially for the complex traits using the core-collection, meaning that as diversity increases, QTL detection power decreases.

In some cases we observed low LD (r^2^<0.01) between a QTL and the significant associations indicated by the best model of mlmm. Some of these markers were found at the same time close to the target QTL and tightly linked to another more significant association. This phenomenon could result from an extremely large QTL effect; as, in addition, the causal loci were not included in the analysis, its variation was thus captured by multiple “complementary” SNPs not completely linked to the QTL. The other part of weakly linked associations was further from the QTL and can be the result of remaining kinship and population structure.

### Prediction of phenotypes from genotypes by GEBV

We will discuss here our GS results focusing on three points: i) the comparison of prediction methods ii) the definition of training and candidate sets in a structured population iii) the influence of trait structure on prediction accuracy.

Several studies identified parameters affecting prediction accuracy. The significance of marker density, size of the training population and trait heritability have already been well assessed [10], [73], [74]. Therefore, we defined our parameters according to these previous findings, adjusting them to grapevine genome in order to reach optimal prediction accuracy: number of polymorphic SNPs (MAF>0.05 filtered) around 81,000 (one SNP in each 5.8 kb), training population size at 1,000, and heritability between 0.7 and 0.8.

#### Prediction methods

We realized genomic predictions on simulated grapevine data using four methods, viz. a classical MAS approach with the cofactors identified in mlmm analysis (cof) and three “all genome” methods: Ridge-Regression BLUP (RR), Bayesian LASSO regression (BLR) and marker assisted Ridge-Regression (cofRR). For the cof and cofRR prediction models, we retained all significant cofactors identified by mlmm, and re-estimated their effects in a mixed model. Our results show that, by considering these effects, higher prediction accuracies can be obtained than by estimating all effects with RR or BLR methods (except for non-structured simple trait predicted with the core-collection on the totality of descendants, where RR, BLR and cofRR were on the same level and cof method outperformed them). The only cofactor-using method (cof) was also as or more efficient than RR and BLR methods in all cases, except for the prediction of the complex trait with the core-collection. A number of authors have shown that there are two major factors affecting prediction accuracy: LD between marker and QTL, and information on the genetic relationship captured by markers [75]–[78].

The cofRR method uses two types of genomic information: i) the associated cofactors identified by GWA approach (mlmm) that capture the accuracy due to LD between marker and QTL, and ii) the remaining markers of the polygenic term that capture the genetic background effect (such as population structure) of the training set. By contrast, cof method is using the first type of information only, while RR and BLR are principally capturing the genetic background effect [75]. The accuracy due to LD between marker and QTL supersedes the accuracy due to genetic relationship if SNP effect and/or LD are high [76], [77], [79]. Our results on simple and complex traits are in agreement with this, i.e. prediction accuracy of cof method was higher in simple traits than in complex traits, where much fewer QTL could be detected by GWAS (in average 32–59% per replicate for simple traits and 2 to 5% for complex traits). On the other hand, cof method was as efficient as RR and BLR even in complex traits that can likely be explained by the proportion of causal loci compared to neutral SNPs. The 100 QTLs of the complex traits represent 0.09% of the simulated loci, which is still far from the hypothesis of RR and BLR methods, that all or most of the markers have an effect different from zero. Moreover, [80] showed that, for a Bayesian prediction model, redundant and uninformative markers diminish prediction accuracy. Finally we can recommend the use of the cofRR method, which was able to predict a large part of the polygenic term, i.e. the variance not captured by the cofactors, even in complex traits.

Tests on pine data confirmed that cofRR outperforms RR when cofactors could be identified in the training panel. However this advantage strongly relies on the quality and efficiency of GWA analysis with mlmm which provides the cofactors. Present results emphasize the importance of marker density – which is a limiting criterion in real data – and information about population structure in the training material.

#### Combination of training and candidate sets

We performed genomic predictions using four training sets and four candidate sets issued from crosses between selected training individuals, comparing four methods on four traits (simple/complex and structured/non-structured). Three of the four training sets (WW, WE, TE) comprised all individuals in each sub-population. The fourth training set (Call) was the core-collection defined from the entire meta-population, in order to maximize diversity using 1,000 individuals, including the founders of the four candidate populations. Predictions were developed either using models trained on the population from which the founders were chosen (within sub-population) or from the other populations (between sub-populations), or on a core-collection representing the diversity of the entire meta-population.

According to [48], lower accuracies were obtained when the training set was not related to the candidate populations (between sub-populations) due to the lower genetic relationship between training and candidate sets. In fact, in our scenario, the three sub-populations diverged from each other due to genetic drift through 500 generations. Differentiation was accelerated by selection and slowed down by migration between sub-populations. However, Figure S9 in Information S1 shows that the effect of QTLs did not vary much between sub-populations, maintaining the accuracy due to LD between marker and QTL. The highest accuracies (up to 0.9) were obtained either in within sub-population predictions or when using the core-collection as training population. Consistent with [81] and [48], the combination of the individuals of all sub-population in the core-collection yielded as good an accuracy as in within sub-population situations. We have to specify here that the high marker density used in this study allowed capturing the effect of multiple polymorphic QTLs and a great part of the genetic relationship even if sub-populations diverged.

#### Influence of trait structure

Our results show that population structure affects prediction accuracy in both simple and complex traits. Globally we observe that non-structured traits were predicted with higher accuracy (Figure 6). However, we observe higher accuracy for structured traits than for non-structured ones when predicting the entire breeding meta-population with all-genome using models (RR, BLR, cofRR) built on the core-collection (accuracy of 0.6 and 0.98 respectively; Figure 7). Therefore, if there is a significant population structure in the training population and in the candidate set, a trait following this structure is better predicted than a non-structured trait. A plausible explication for these results is that, in contrast to cof method, RR and BLR methods could capture the population structure in the core-collection. This becomes advantageous when the candidate set displays that same population structure (with all groups of structure), and leads to supplementary knowledge in the case of traits which co-segregate with this structure.

In conclusion, we can recommend the use of the cofRR method, which makes simultaneous use of information about QTLs (through cofactors obtained from GWAS), genetic relationship and population structure. Contrary to GWAS, GS using either RR, BLR and cofRR methods is able to take advantage of the population structure when predicting structured traits, if both training and candidate populations are following the same pattern.

This work is the first attempt to test both GWAS and GS in grape through simulations. On a large population of 3,000 individuals, up to 81,555 SNP markers with frequency above 5% and four traits (simple and complex, structured and non-structured) were simulated. Through GWAS, an average of 5.9 to 30% of the QTLs could be identified, the best results being obtained for simple non-structured traits. Genomic estimated breeding values (GEBV) were calculated using the same data set. Predictions for simple traits within population were always more accurate, with a very high accuracy of 0.9, while accuracy dropped to 0.2 for complex trait and betweenpopulation predictions. Accuracy also depended on the pairs of populations in relation with the mean phenotypic differences between the training and candidate populations. The highest prediction accuracy (up to 0.9) was obtained using the combined GWAS-GS model (cofRR). Finally, for grapevine breeding or for other important economic crops with the same characteristics, we recommend using the combined prediction model with a core-collection as training population.

## Supporting Information

Information S1 List of the supplementary figures, files and tables.
(PDF)
