# Supplementary material for: Genome-Wide Prediction Methods in Highly Diverse and Heterozygous Species: Proof-of-Concept through Simulation in Grapevine
Source: PLoS One. 2014 Nov 3;9(11):e110436. doi: 10.1371/journal.pone.0110436 (PMC4217727; doi:10.1371/journal.pone.0110436)
Supplement: Information S1 — List of the supplementary figures, files and tables. (PDF) [file pone.0110436.s001.pdf]

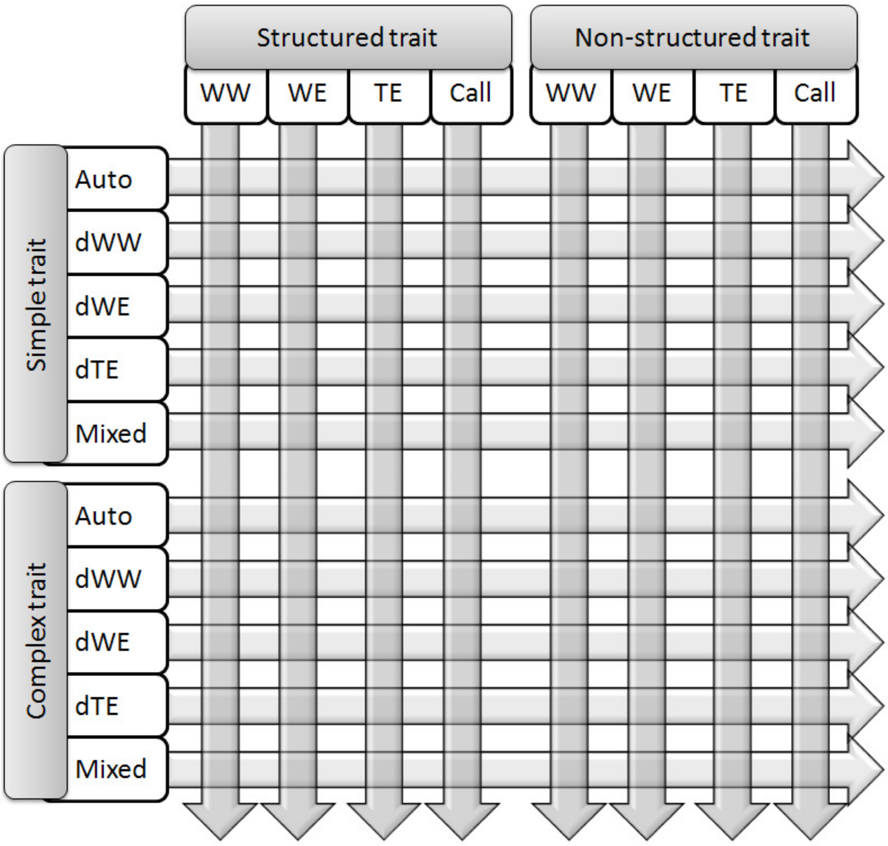

Figure S1 Experimentation plan to study the evolution of the precision in different combinations of training and candidate population, at two levels of genetic architecture's complexity (10 or 100 underlying QTLs) and predicting a structured and a non-structured trait. For each possibility 4 prediction methods were used: sum of cofactors of MLMM (cof) Ridge Regression (RR), Bayesian Ridge Regression (BRR) and a marker assisted RR-BLUP (cofRR).

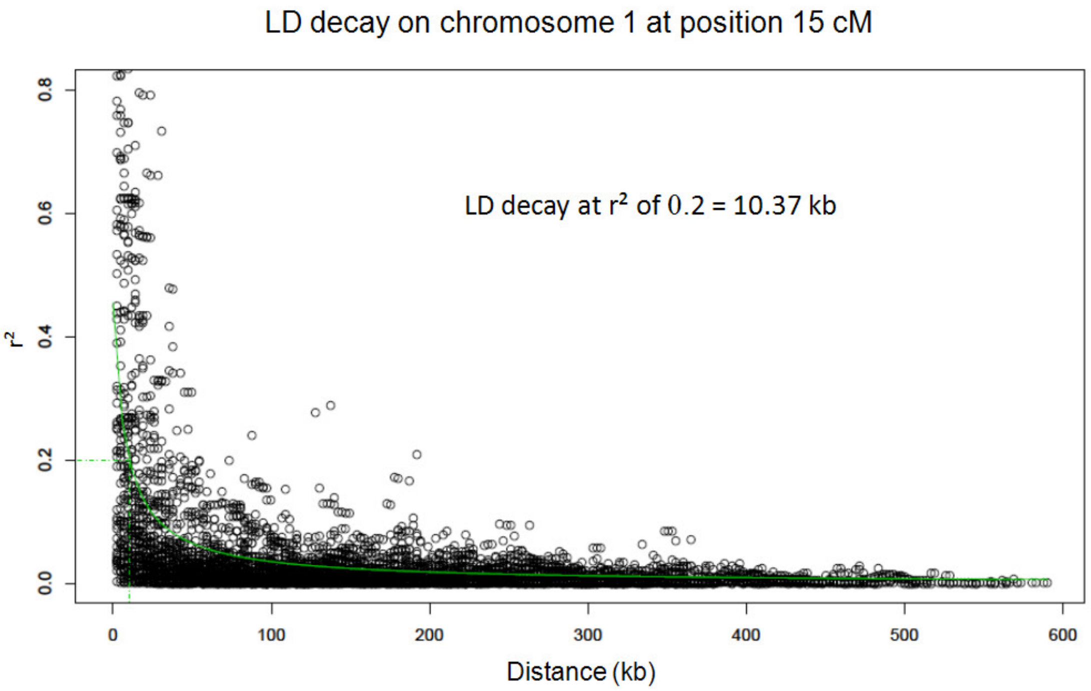

Figure S2 LD decay between each pair of SNP in a 600 Kb window of a neutral region (around the position 15 cM on the first chromosome) of one simulated replicate. Measures were performed with r^2_{SV} on 3000 individuals The green line represents the LD decay (HILL and WEIR 1988) estimated from the raw data (black point).

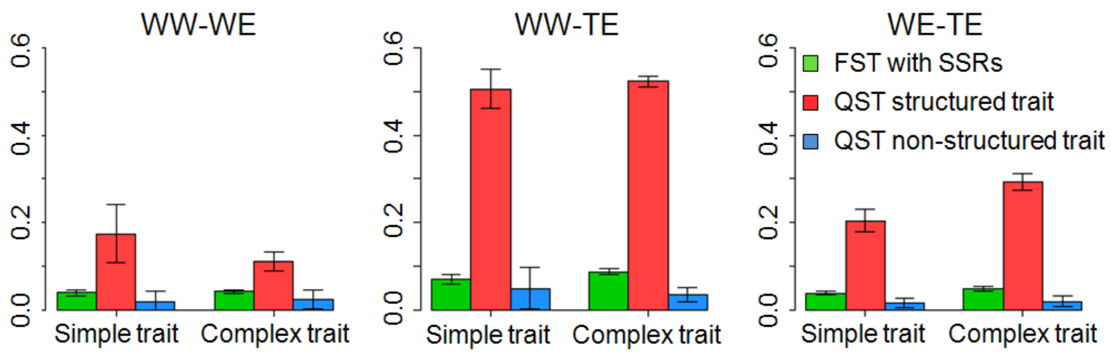

Figure S3 Diversity indices of the simulated data: The mean F_{ST} and Q_{ST} between the three simulated populations (WW-WE-TE) calculated on selected and non-selected traits through 10 replicates for both simple and complex traits. Error bars were calculated with 95% confidence intervals on the estimates of the means.

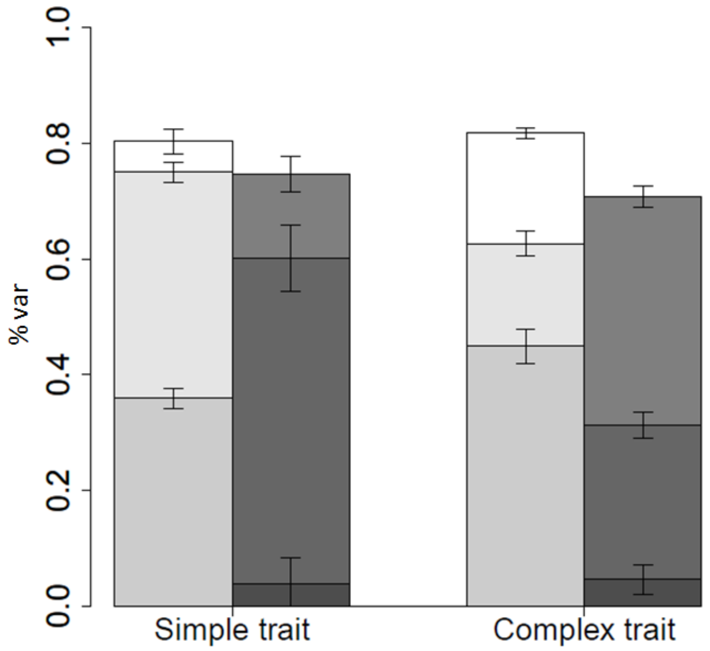

Figure S4 Composition of the variance explained with the best model of mlmm. Results are showed for the structured and non-structured simple and complex traits on 10 replicate of the simulation. The first bars represent structured traits and the second ones represent non-structured traits. The darker color is the part explained by population structure, the intermediate color show the part of cofactors and the lightest represent the part of the polygenic term. To model selection we used mBonf criterion.

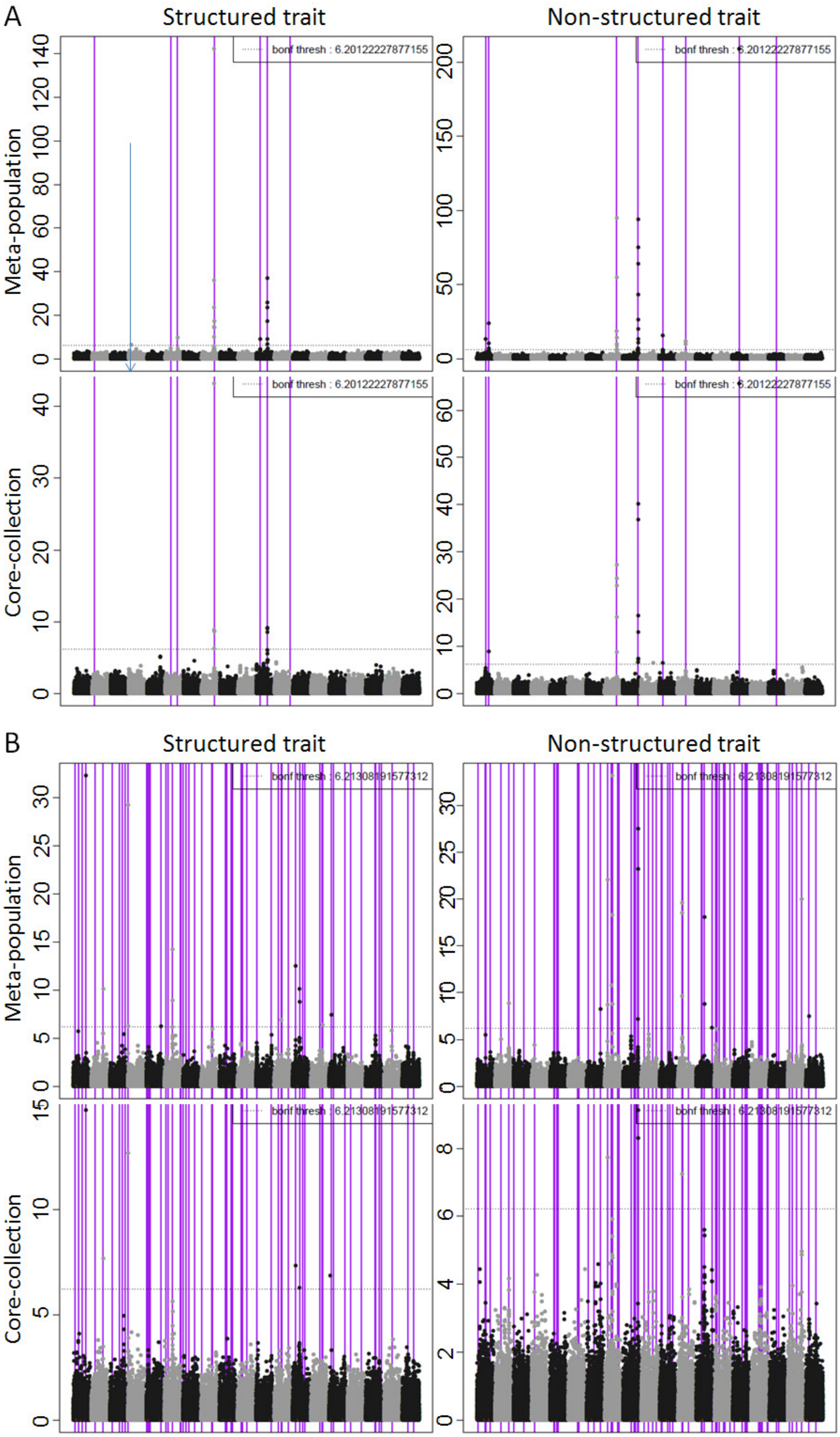

Figure S5 Manhattan-plots of GWAS performed with mlmm on one replicate of the simulation. (A) presents the results for simple (10 QTLs) structured and non-structured trait on the core-collection of 1,000 individuals and on the entire meta-population (3,000 individuals). (B) part presents the results for complex traits (100 QTLs). Violet bars represent QTL loci with MAF>0.05, blue bars are QTLs with MAF<0.05.

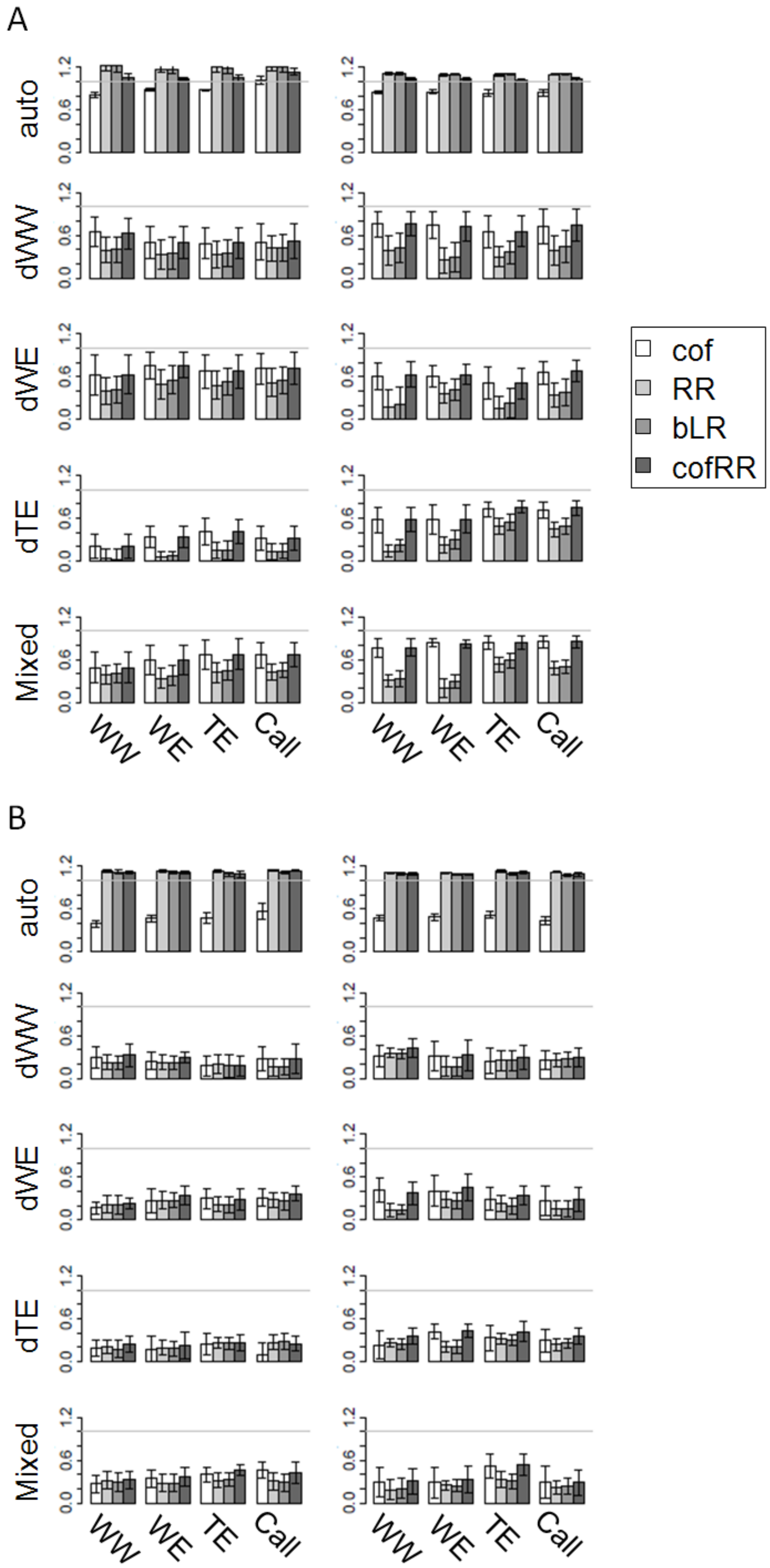

Figure S6 The accuracy of the prediction through different combinations of training and candidate population. At the left side we show structured trait and on the right side the non-structured one. Colors represent the four prediction methods used: the sum of cofactor's effects identified in MLMM ("cof"), Ridge Regression BLUP ("RR"), Bayesian LASSO ("BLR") and marker assisted RR ("cofRR"). We used the three simulated populations (WW, WE, TE) and the entire core-collection (Call) as training population and realized prediction on the same sample (auto) and on each training sup-population (dWW, dWE, dTE, Mixed). On the left side we present the structured trait and on the right side the non-structured one. (A) presents the results on the simple trait (10 QTLs). (B) presents the complex trait (100 QTLs).

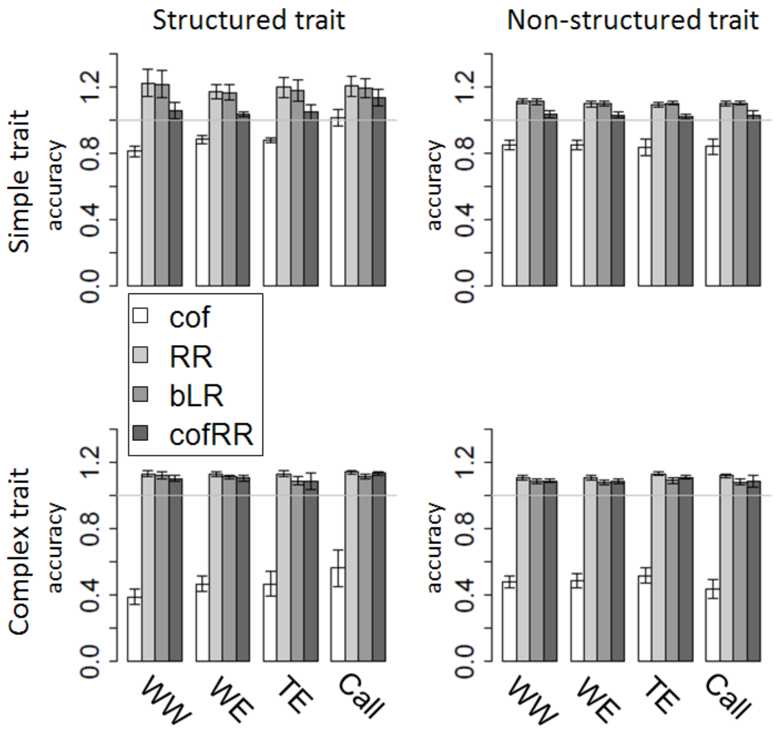

Figure S7 The accuracy of auto-prediction on each training set (WW, WE, TE, Call) for all traits (structured / non-structured and simple or complex) with all four implemented methods (cof, RR, BLR, cofRR).

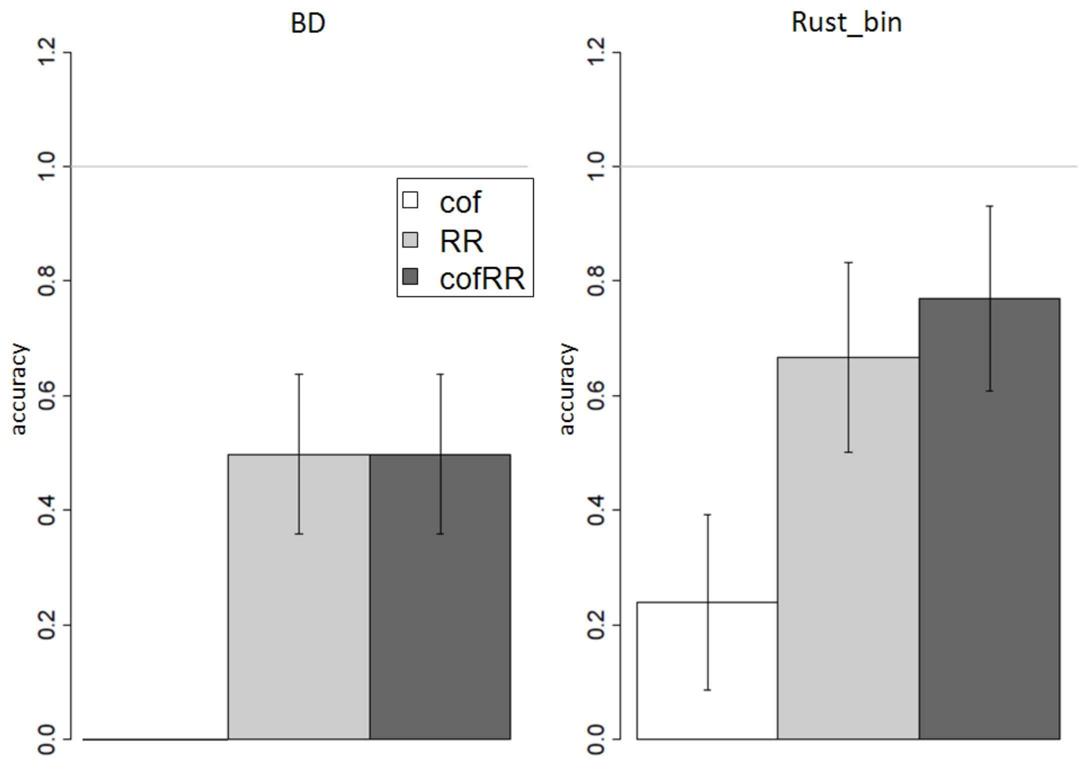

Figure S8 Prediction accuracy for two traits of the pine data using cof, RR and cofRR methods. BD: average branch diameter of six years old trees; Rust_bin: fusiform rust susceptibility by presence or absence of rust. Error bars were calculated with 95% confidence intervals on the estimates of the means.

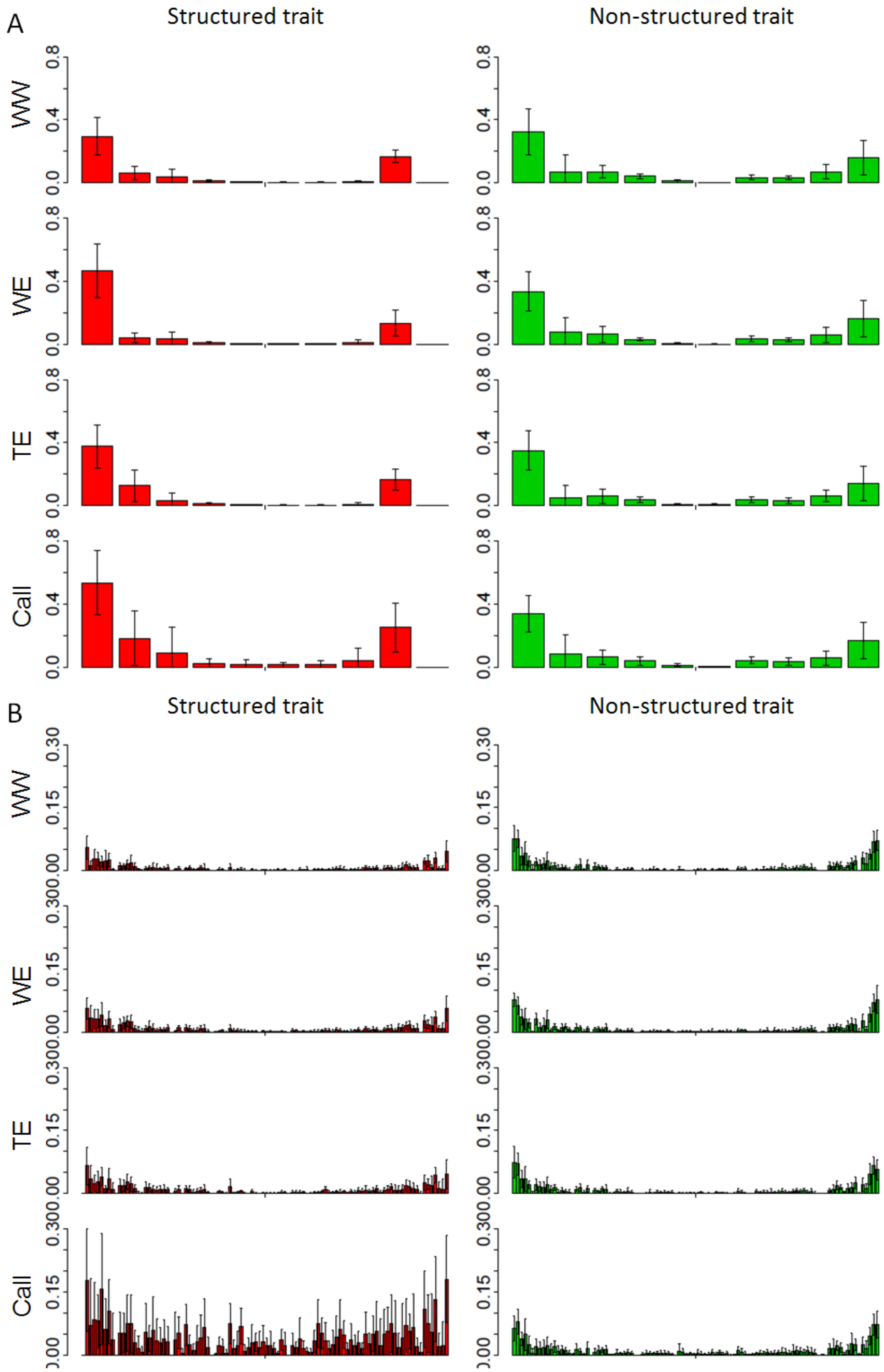

Figure S9 The percentage of the variance explained by each QTL, measured separately on the three sub-population (WW, WE, TE) and on the core-collection (Call). Red bars are structured traits QTLs and greens are non-structured trait's QTLs. (A) represents simple traits (10 QTLs). (B) complex traits (100 QTLs).

File S1

GrapeSim.RData

File S1 is available for download as RData [.RData] at https://www.dropbox.com/s/x3esk4go6b34713/GrapeSim.RData

This file contains five R objects:

- X: a n by m matrix, where n=number of training individuals, m= number of SNPs, with rownames(X)=individual names, and colnames(X)=SNP names
- Xv: a nV by m matrix, where nV=number of validation individuals, m= number of SNPs, with rownames(Xv)=individual names, and colnames(Xv)=SNP names
- Y_ok: vector of phenotypes of the training set: a vector of length n, with names(Y_ok)=individual names
- Yv_ok: vector of phenotypes of the validation set: a vector of length nV, with names(Yv_ok)=individual names
- PC: a n by k matrix, where m= number of individuals, k= number of groups/PCA axes, with rownames(PC)=individual names colnames(PC) name of groups

File S2

COFRR_FODOR ET AL.R

File S2 is available for download as an R script [.r] at https://sites.google.com/site/vincentosegura/cofrr

Table S1 Results of the GWA performed on the replicate N°25.

		structured trait							structured trait						
		Association sign.		max -log10(P)	max var.expl	detected QTL	distance (kb)	r^2_{SV}	Association sign.		max -log10(P)	max var.expl	detected QTL	distance (kb)	r^2_{SV}
		all	r^2>=0.05						all	r^2>=0.05					
Simple trait	meta-population	16	11	142.35	0.26	4	0-201	0.05-0.62	20	13	208.99	0.146	6	2-125	0.05-1
	core-collection	7	5	43.22	0.145	2	9-106	0.17-0.55	16	12	65.69	0.125	4	0-125	0,08-1
Complex trait	meta-population	13	10	32.3	0.052	10	2-135	0,06-0.66	19	4	27.54	0.033	3	0-132	0,15-0.36
	core-collection	6	5	14.71	0.027	5	4-149	0.1-0.72	4	2	9.13	0.029	1	9-45	0,2-0.33

Analyses were on the 3,000 individuals of the meta-population and the 1,000 individuals of the core-collection. Association was considered if the p-value passed the 5% Bonferroni threshold. The maximum of the –log(P-value), the variance explained by the SNP, the number of detected QTLs, the distance and the r^2_{SV} between significant SNP and QTL, were presented only for the associations where the r^2_{SV} between SNP and QTL was at least 0.05.
